# Future changes in hydro-climatic extremes in the Upper Indus, Ganges, and Brahmaputra River basins

**DOI:** 10.1371/journal.pone.0190224

**Published:** 2017-12-29

**Authors:** René R. Wijngaard, Arthur F. Lutz, Santosh Nepal, Sonu Khanal, Saurav Pradhananga, Arun B. Shrestha, Walter W. Immerzeel

**Affiliations:** 1 FutureWater, Costerweg 1V, Wageningen, The Netherlands; 2 Utrecht University, Department of Physical Geography, Utrecht, The Netherlands; 3 International Centre for Integrated Mountain Development, Khumaltar, Kathmandu, Nepal; Universidade de Vigo, SPAIN

## Abstract

Future hydrological extremes, such as floods and droughts, may pose serious threats for the livelihoods in the upstream domains of the Indus, Ganges, Brahmaputra. For this reason, the impacts of climate change on future hydrological extremes is investigated in these river basins. We use a fully-distributed cryospheric-hydrological model to simulate current and future hydrological fluxes and force the model with an ensemble of 8 downscaled General Circulation Models (GCMs) that are selected from the RCP4.5 and RCP8.5 scenarios. The model is calibrated on observed daily discharge and geodetic mass balances. The climate forcing and the outputs of the hydrological model are used to evaluate future changes in climatic extremes, and hydrological extremes by focusing on high and low flows. The outcomes show an increase in the magnitude of climatic means and extremes towards the end of the 21^st^ century where climatic extremes tend to increase stronger than climatic means. Future mean discharge and high flow conditions will very likely increase. These increases might mainly be the result of increasing precipitation extremes. To some extent temperature extremes might also contribute to increasing discharge extremes, although this is highly dependent on magnitude of change in temperature extremes. Low flow conditions may occur less frequently, although the uncertainties in low flow projections can be high. The results of this study may contribute to improved understanding on the implications of climate change for the occurrence of future hydrological extremes in the Hindu Kush–Himalayan region.

## Introduction

The Hindu Kush–Himalayan (HKH) region plays a crucial role in the South Asian hydrology [1,2]. It encompasses the headwaters of the Indus, Ganges and Brahmaputra (IGB), and supports the livelihoods of about 700 million people living in these basins [3,4]. It sustains the seasonal water availability by means of meltwater originating from upstream ice and snow reserves and it supplies water that is utilized for agriculture (e.g. irrigation), energy production (e.g. hydropower), industry, navigation, and drinking water supply [5–8]. There is a growing concern that the hydrology in the IGB might be affected by future climate change. In the last few decades, several studies (e.g. [2,7,8]) have outlined that future climate change will affect the hydrological regimes in the IGB. It is likely that rising temperatures and precipitation changes will affect glacier volumes, seasonal snow cover, and runoff characteristics, and thus the water availability in both up- and downstream parts of the IGB [9–11]. Likewise, it is expected that the frequency and magnitude of extreme hydrological events (i.e. the occurrence of floods and droughts) will rise, posing serious threats for the livelihoods of people living in the IGB [12–14].

Many studies have documented evidences of historic climate change in the IGB. Among the observed trends in climatic variables, the increasing temperature trend is most consistent over the region. For instance, in parts of the Upper Ganges Basin (Nepal) temperature have increased at a rate of 0.06°C y^-1^ between 1978 and 1994, with higher rates at higher elevations [15]. In the Upper Brahmaputra Basin, the average annual temperature has increased at a rate of 0.03°C y^-1^ between 1961 and 2005 [16], whereas in the Upper Indus Basin, both, increasing and decreasing temperature trends have been observed since the 1960s. Thereby, the decreasing temperature trends were attributed to the decline in mean summer temperature [17]. Precipitation trends that have been reported in the HKH region show mixed signals with increasing precipitation trends in the western part of the HKH [3,18], and no distinct trends in other parts of the HKH [2,15,19].

Future climate change will likely be associated with a continued warming over the 21^st^ century. Temperature increases between 1.7°C and 6.3°C are projected towards the end of the 21^st^ century in the IGB, where elevation-dependent warming will likely result in stronger temperature increases in the mountainous regions of the HKH than in the adjacent lowland regions [20–22] as observed in the historical temperature trends [15]. Based on the projected increases, accelerated melt rates can be expected until the mid of the 21^st^ century, thereby affecting stream flow. In the second half of the 21^st^ century melt water rates are projected to decline in the HKH. Stream flow is however still projected to increase by then, which can mostly be attributed to increases in precipitation [23].

In general, future precipitation is projected to increase in the upstream basins of the IGB [9,19,24–27]. In addition, there are also parts of the HKH region (e.g. the northwestern part of the upstream Indus basin) where precipitation is projected to decrease [14]. The confidence in future precipitation projections is however low due to the large spread in future projections and the model’s limitations to simulate complex mountainous climates of South and Central Asia [20,28]. Based on the projected precipitation changes, it is likely that both droughts and floods will occur more frequently into the future. According to a previous report [1] it is likely that droughts will occur more often in the Indus basin, thereby having consequences for the food production. The projected precipitation increases are likely to result in higher peak flows and associated risks for flood hazards. The Pakistan floods of July-September 2010, caused by intense monsoonal rainstorms that penetrated unusually far in the Himalaya and the Karakoram [29], illustrate the devastating impact floods can have on a society. The floods resulted in about 1950 fatalities, an estimated overall loss of 9.5 billion US dollars, and affected about 20 million people [1,30,31]. Considering economic and population growth in the flood-prone areas of the IGB (e.g. Bangladesh), losses and the number of fatalities could be even larger in the future [12,32]. The (potential) impacts of droughts and floods on the livelihoods in the IGB illustrate the vulnerability to hydrological extremes.

In recent years, many climate impact studies have so far mainly focussed on the impacts of climate change on hydrological regimes in, both, large and small river basins [2,6,9,26,33–35]. These studies have shown that future water availability will likely be sustained over the 21^st^ century. Some studies have also indicated that hydrological extremes pose a larger threat. The problem however is that knowledge about future changes in hydrological extremes resulting from climate change is lacking in the IGB. Few studies have been conducted on the explicit effects of climate change on hydrological extremes in the IGB. For instance, a recent study, assessing the impacts of climate change on hydrological regimes and extremes in the Upper Indus Basin, showed that, in general, summer peak flow will likely shift to other seasons, and projected an increase in the frequency and intensity of extreme discharge conditions [14]. Another study projected increases in heavy precipitation indices during monsoon period, accompanied with extended periods of no precipitation during the winter months, in the Ganges basin [36]. Hence, the cited study [36] indicated an increase in the incidence of extreme weather events over the first half of the 21^st^ century. Studies performed on global flood risk show similar patterns [13,37,38]. Significant increasing trends in high flows (i.e. 10 percentile exceedance discharge) were found in the Ganges basin with relative increases up to about 100% [37]. Thereby, the changes in high flows were projected to be more significant than the changes in low flows (i.e. 90 percentile exceedance discharge). Assessments on future flood and drought frequencies in a number of basins spread across the world, including the IGB basins, found that, in the Ganges and Brahmaputra river basins, a future 100-year flood (i.e. equivalent to discharges with a 100-year return period in the 20^th^ century (1901–2000)) will occur once in 26.1 years and 3.8 years, respectively, at the end of the 21^st^ century [38]. Furthermore, the average number of drought days were found to increase by a factor 1.17 and 4.05 in the Ganges, and Indus basins, respectively [38]. Most of the studies regarding hydrological extremes have focussed on both up- and downstream parts of the IGB without considering the effects of climate change on hydrological processes that are relevant in mountainous basins, such as snow and ice melt. For this reason, an improved understanding is needed on the impact of climate change on these processes and their implications for the occurrence of hydrological extremes in mountainous basins.

The representation of future hydrological extremes is highly depending on the representation of climatic extremes in General Circulation Models (GCMs) that force the hydrological models. Previous studies [39,40] investigated the performance of GCMs from the Coupled Model Intercomparison Project 5 (CMIP5) in simulating climatic extremes. These studies show that the climate models are generally able to simulate climatic extremes and their trend patterns, and that the spread among different climate models for several temperature indices has reduced in comparison with CMIP3 models, despite the larger number of CMIP5 models. In addition, the representation of precipitation extremes has also improved. Nevertheless, there is still some discrepancy in the simulation of some precipitation indices. Further it is shown that the analysed CMIP5 models generally agree on the projected trends in temperature extremes. However, in some regions, such as South Asia, there is no consensus between GCMs on projected trends in a few precipitation indices, such as consecutive dry days (CDD). Some of the models project an increase in CDD, whereas others project a decrease in CDD. Similar contradictive projections were also found for the Indus, Ganges, and Brahmaputra, whereas other indices were in line with projected trends in climate extremes, though the spread between the models can be large [20]. The presence of discrepancies between GCMs emphasizes the importance of selecting climate models, based on the model’s skill to simulate climatic extremes in regions of interest, for the assessment of hydrological extremes.

The main aim of this study is to investigate the impacts of climate change on future hydrological extremes in the upstream domains of the Indus, Ganges, and Brahmaputra basins. To this end, we apply a fully distributed cryospheric-hydrological model. The model is forced with the outputs of 8 GCMs (i.e. representing RCP4.5 and RCP8.5) that were pre-selected by using an advanced envelope-based selection approach [20]. Subsequently, the outputs of the hydrological model are analyzed on hydrological extremes by focusing on high and low flows. The novelty of this study in comparison with previous work in the region (e.g. [14]) is that it is first to investigate the full range of possible impacts of climate change (i.e. in terms of climate extremes) on the occurrence of both high and low flows in the upstream mountainous domains of the entire IGB. Most studies that have been conducted in the IGB only focused on the downstream parts of the IGB or in the entire IGB (e.g. [13,41]) and did not take processes into account that are relevant in mountainous basins (e.g. ice and snowmelt). In the upstream domains of the IGB, where mountain-hydrological processes are important, the number of studies on extremes is very limited. To our knowledge, a previous study conducted in the upstream Indus basin [14], is so far, the only study on hydrological extremes at this scale, and takes mountain-hydrological processes into account. Nevertheless, the cited study [14] is only about high flows and does not take the effects of climate change on low flows into consideration. For this reason, our study contributes to an improved understanding on the effects of climate change on both high and low flows in the mountainous domains of the Indus, Ganges, and Brahmaputra.

## Study area

**Fig 1:**
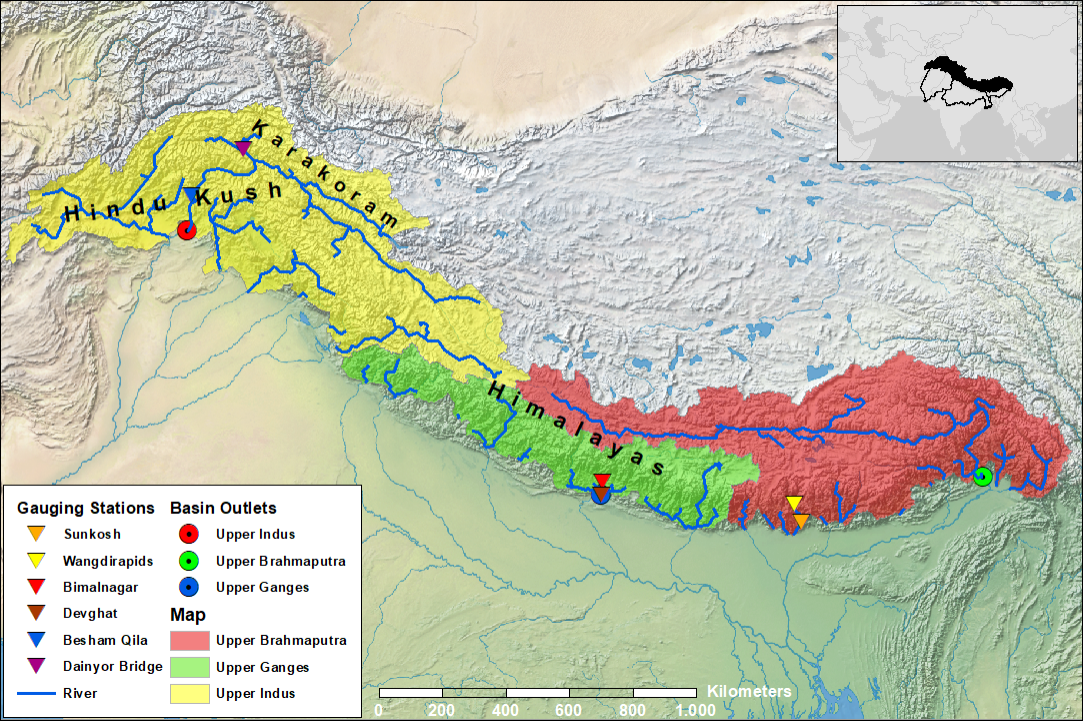
Study area. Map of the study area showing the outlets of the basins and the gauging stations used for calibration and validation of the model. Source of the background basemap imagery and the political borders displayed in the inlet of the figure is www.naturalearthdata.com.

Future changes in hydrological extremes are assessed for the upstream parts of three major river basins with origins in the Hindu Kush—Himalaya (HKH): the Upper Indus Basin (UIB), the Upper Ganges Basin (UGB), and the Upper Brahmaputra Basin (UBB) (see Fig 1). The Upper basins are defined as the areas that extend from the sources of the Indus, Ganges and Brahmaputra to the northern margins of the Indo-Gangetic plains. The UIB, UGB, and UBB cover a surface area of about 399,000 km^2^, 168,000 km^2^, and 370,000 km^2^, respectively, in the HKH mountain ranges. The altitude ranges from 8850 m above sea level (a.s.l.) in the UGB to about 100 m a.s.l. at the southern margins of the UBB. Glaciers cover a total surface area of about 21,000 km^2^, 9,000 km^2^, and 14,000 km^2^ in the UIB, UGB, and UBB, respectively [42].

**Fig 2:**
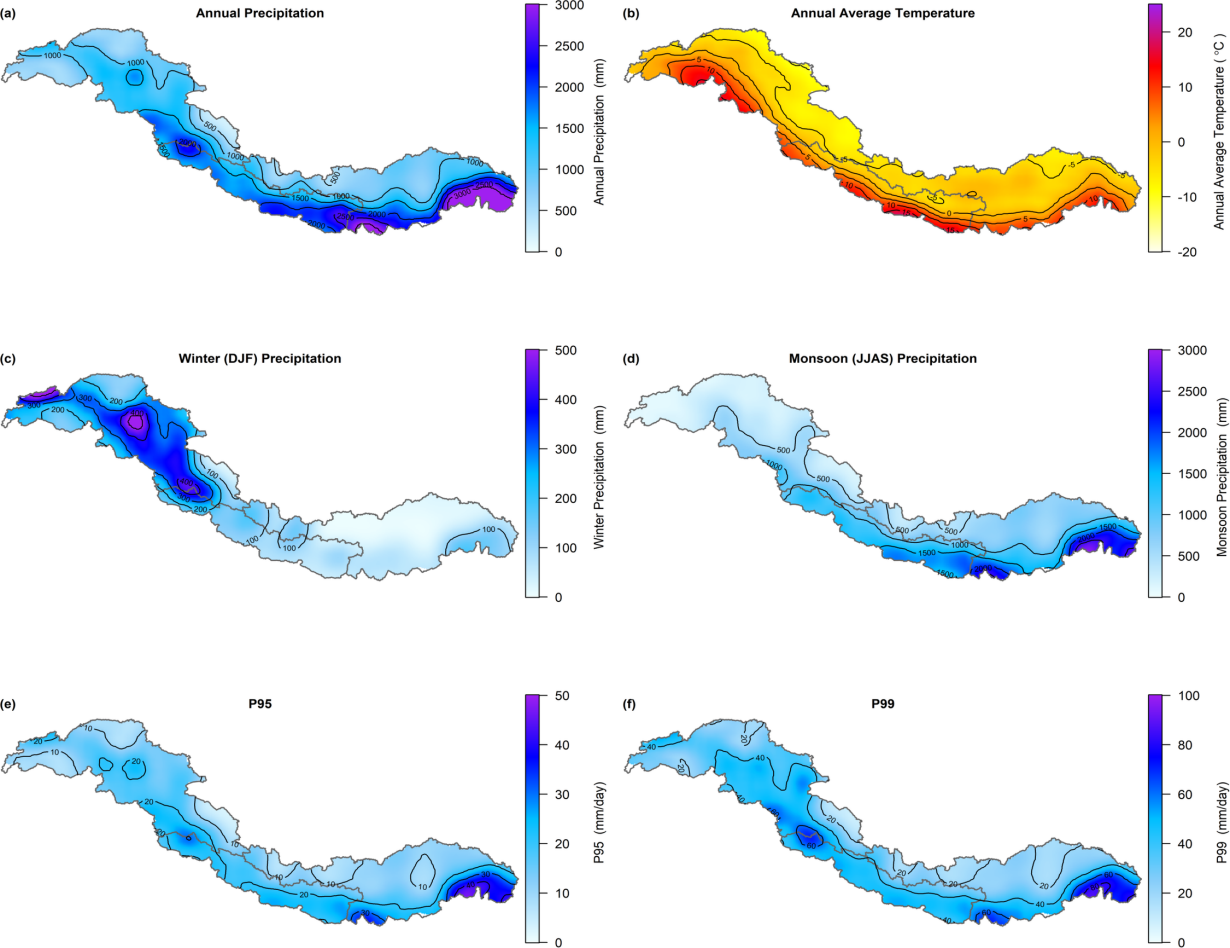
Climate of the Upper Indus, Ganges, and Brahmaputra basins. Maps of the upstream domains of the Indus, Ganges, and Brahmaputra showing the annual precipitation (a), the average air temperature (b), the winter precipitation (c), the monsoon precipitation (d), and the precipitation extremes (P95 (e) and P99 (f)) for the reference period. Abbreviations: DJF = December, January, February, JJAS = June., July, August, September, P95 = 95^th^ percentile of daily precipitation sums, P99 = 99^th^ percentile of daily precipitation sums. Source of the maps is a reference climate dataset [45].

The climate of the upstream domains of the IGB is dominated by the East Asian and Indian monsoon systems, and the Westerlies. The influence of the East Asian and Indian monsoon systems is generally largest in the eastern part of the Himalayas. In these regions most precipitation occurs during the period June-September (see Fig 2D), and orographic effects result in a strong north-south gradient in precipitation intensities [43]. More to the west, the westerlies become increasingly important. In the Hindu Kush and Karakoram, precipitation is more equally divided over the year due to the influence of both the westerlies in the winter and the monsoon systems in the summer [44]. In the Karakoram and at the western margins of the UIB most precipitation occurs during the winter period (see Fig 2C). Annual precipitation sums in the entire IGB ranges from ~100 mm at the Tibetan Plateau to ~5500 mm at the southern margins of the UBB (see Fig 2A). In the latter area, also the precipitation extremes (i.e. 95^th^ (P95) and 99^th^ (P99) percentiles of daily precipitation sums) are largest (see Fig 2E and 2F). The annual average temperature is highest at the southern margins of the UGB with ~24°C and lowest in the high-altitude regions of the Karakoram with ~-19°C (see Fig 2B).

The hydrological regimes that dominate in the IGB differs per region. In the UIB, a glacial melt dominated regime prevails with a glacier melt contribution of 40.6% to the total runoff [9]. In the UGB and UBB, rainfall dominated regimes prevail with a slightly higher contribution of snow- and glacier melt in the UBB [9].

## Data and methods

### Cryospheric-hydrological modelling

We use the physically-based fully-distributed Spatial Processes in Hydrology (SPHY) cryospheric–hydrologic model [46] to simulate current and future daily discharge in the upstream domains of the Indus, Ganges, and Brahmaputra. The model is set up at a spatial resolution of 5 x 5 km and reports on a daily time step.

Daily discharge is simulated by a) calculating total runoff for each grid cell, consisting of four different runoff components: glacier runoff, snow runoff, rainfall runoff (i.e. the sum of surface runoff and lateral flow), and baseflow, and b) routing the total runoff and its components downstream, using a simplified routing scheme that requires a digital elevation model (DEM) and a recession coefficient. The total runoff (Q_TOT_) is calculated for each time step by:

$$Q_{TOT}=Q_{GM}+Q_{SM}+Q_{RR}+Q_{BF}$$ (1)

where Q_GM_ (mm) is glacier runoff, Q_SM_ (mm) is snow runoff, Q_RR_ (mm) is rainfall runoff, and Q_BF_ (mm) is baseflow. For the estimation of the contribution of glacier runoff, sub-grid variability (i.e. on 1 x 1 km resolution) is taken into account. The sub-grid variability is determined by fractional ice cover where fractional ice cover ranges between 0 (no ice cover) and 1 (complete ice cover). In addition, a unique identifier is created for each glacier, or a part thereof, within a model cell. This unique identifier is used for the attribution of information, such as glacier mean elevation, initial ice-thickness, and the type of glacier (i.e. debris-free or debris-covered). Hence, the type of glacier is determined by the differentiation between debris-covered and debris-free glaciers, which is based on thresholds for slope and elevation [47]. Initial ice thicknesses are estimated according to a methodology that has been described in previous studies [23,33]. Glacier melt is calculated according to a degree-day approach [48], where different factors are applied on debris-free and debris-covered glaciers. The produced melt is subsequently subdivided over the surface runoff and baseflow pathways by a calibrated glacier runoff fraction.

To model future changes in the fractional glacier cover, the SPHY model is modified by improving the existing glacier module. In the former glacier module, glaciers were implemented as fixed masses, which could change over time using a parameterization for glacier changes at the large river basin scale [49]. This approach has no consideration of mass conservation and ice redistribution. In the improved glacier module, these processes are included. A more detailed description regarding the improved glacier module of the SPHY model has been published before [50].

For those parts of the cells that are not covered by glaciers, a dynamic snow storage is simulated according to a degree-day snow model [51]. The snow accumulation and–melt is simulated by a degree-day approach similar to the approach that is used to simulate glacier melt. Snow sublimation is estimated by a simple elevation-dependent potential sublimation function [14]. This function assumes potential sublimation to increase linearly with elevation above 3000 m a.s.l. by a calibrated sublimation factor and presumes the majority of sublimation to originate from snowblown sublimation, thereby assuming that the highest wind speeds and driest air conditions prevail at the higher altitudes. The actual sublimation is limited by total snow storage within the grid cell. In addition to snow melt, accumulation, and sublimation, refreezing of snowmelt and rain are included as well. When snow cover is absent, rainfall runoff processes are simulated where a part of the rain is transported directly into the river network by surface runoff, and another part is transported to the network via lateral flow from the soil water storage or baseflow from the groundwater storage. For the simulation of soil water processes, processes as evapotranspiration, infiltration, and percolation are included. These processes are simulated for a topsoil and subsoil layer. A more detailed description of the SPHY model has been published before [46].

### Datasets

As meteorological forcing, we use a dataset of daily air temperature and precipitation fields at 5 x 5 km resolution developed for the Indus, Ganges and Brahmaputra river basins [45]. This dataset is based on the Watch Forcing ERA-Interim (WFDEI) dataset [52]. The raw temperature data are spatially interpolated (i.e. by using a cubic spline interpolation) from a resolution of 0.5° x 0.5° to a resolution of 1 x 1 km, and subsequently downscaled using a 1 x 1 km digital elevation model (DEM) and vertical monthly temperature lapse rates. The downscaled temperature data are bias-corrected to the observations of 40 meteorological stations located in the study area. The downscaled temperature data are bias-corrected to the observations of 40 meteorological stations located in the study area. To correct for elevation differences, temperature values were lapsed from station elevation to grid cell elevation using a constant temperature lapse rate of -0.0065°C m^-1^. Long-term mean biases between the gridded product and station data at the station’s locations were interpolated spatially to generate a spatial correction grid, which was applied to the uncorrected temperature fields. In addition, a temperature bias-correction is conducted, by capping the average annual glacier ablation to a maximum plausible value [33,53], to avoid unrealistic high temperatures at high altitudes. The raw precipitation data are spatially interpolated by means of a cubic spline interpolation too, and are subsequently corrected by using geodetic mass balances as a proxy to reconstruct precipitation amounts [54]. Finally, the corrected 1 x 1 km temperature and precipitation datasets are resampled to a resolution of 5 x 5 km (i.e. the model resolution).

High-altitude precipitation is often highly uncertain, due to lacking high-altitude observations and the insufficiency of gridded precipitation products, such as ERA-Interim [55] and APHRODITE [56], in capturing the spatial variation and magnitude of high-mountain precipitation [54]. This is mainly caused by the poor coverage of precipitation gauging stations and limited detection of snow, which eventually results in significant underestimation of precipitation [27,57]. For example, previous work [54] showed by reconstructing required precipitation amounts to sustain observed glacier mass balance and observed discharge that precipitation in the Upper Indus Basin is underestimated by ~200% in regularly used precipitation products, and that locally even ten times the amount of precipitation reported in gridded products would be more realistic. For this reason, spatial precipitation fields usually need correction for the simulation of reliable water balance components. Earlier validation of precipitation fields to observed discharge and estimates of actual evapotranspiration at several gauging stations [45] indicates that precipitation corrections are necessary due to the underestimation of precipitation at most gauging stations. To this end, a precipitation correction factor (i.e. 1.3 in the upstream domains of the IGB, with exception of the Tarbela basin (upstream of Besham Qila) where a factor of 0.85 is used) is applied, which is estimated as the relative difference between observed discharge and simulated discharge resulting from initial model runs with SPHY. The precipitation corrections are applied on the elevation zone between 4500 m a.s.l. and 5500 m a.s.l., which covers the steepest part of the hypsometry in the upstream domains of the IGB, and where the precipitation bias is largest [54,58]. Below 2000 m a.s.l. and above 7000 m a.s.l. no correction is applied. In other elevation zones, linear relations between elevation and correction factors are used to estimate the magnitude of the correction factor.

The meteorological data required for the bias-correction of the temperature datasets are obtained from 40 meteorological stations that are acquired through Nepal Department of Hydrology and Meteorology (DHM), the Pakistan Meteorological Department (PMD), and the Pakistan Water and Power Development Authority (WAPDA). Time series of observed daily discharge from 6 gauging stations are provided by the Bhutan Department of Hydro Met Services (BDHMS), WAPDA, and DHM.

As DEM, we use the 15 arc-second HydroSHEDS DEM [59], which is a void-filled and hydrologically conditioned DEM based on the SRTM DEM [60]. The digital elevation model is resampled to 5 x 5 km resolution. Land use information is extracted from the MERIS Globcover product [61] and soil information is derived from HiHydroSoil [62], which is a high-resolution soil map of hydraulic properties. This map has been derived from the Harmonized World Soil Database [63] using pedotransfer functions. Glacier outlines are derived from the Randolph Glacier Inventory v5.0 [64] and are recalculated to a fractional ice cover on a 1 x 1 km grid. MODIS snow cover data [65,66], IceSat-derived zonal glacier mass balances [67], and discharge time series are used for the model calibration.

### Calibration and validation

The calibration and validation of the SPHY model is performed by using a two-step systematic approach to minimize equifinality problems (e.g. [68]), which are a common problem in the simulation of high-mountain hydrology. Because of the common underestimate of high-mountain precipitation in meteorological forcing products, the water deficit is often compensated by unrealistic high ice melt rates to compensate for this, when calibrated to observed discharge. We followed two steps by first calibrating the snow and glacier parameters to observed snow cover and glacier mass balances, to ensure realistic parameter values for the model parameters related to cryospheric processes. Secondly, we calibrated the rainfall-runoff parameters to observed discharge. After the calibration of all parameters the model is validated to observed discharge. Plausible parameters ranges, used for the calibration of the parameters, are based on a previous report [9] and a local One-At-A-Time (OAT) sensitivity analysis [69] that is conducted prior to the calibration. The parameter ranges are summarized in Table 1.

**Table 1:** Parameters and their ranges used for the calibration.

Parameters	Description	Units	Range
*Glacier*			
DDFG	Degree-day factor debris-free glaciers	mm °C day^-1^	3–9
DDFDG	Degree-day factor debris-covered glaciers	mm °C day^-1^	1–7
*Snow*			
Tcrit	Critical temperature	°C	-3–3
SnowSc	Water storage capacity of snow pack	mm mm^-1^	0–1
DDFS	Degree-day factor snow	mm °C day^-1^	3–9
Subl3Rate	Sublimation rate	mm day^-1^	0–10
*Rainfall-Runoff*			
alphaGw	Baseflow recession coefficient	-	0.001–0.2
deltaGw	Groundwater recharge delay time	day	1–180
kx	Routing recession constant	-	0.01–0.99
GlacF	Glacier melt runoff factor	-	0–1
Rootdepth	Thickness of root zone	mm	50–1000
Subdepth	Thickness of subsoil	mm	300–3000

The parameters related to glacier melt, snow accumulation, and–melt (*DDFG, DDFDG, Tcrit, SnowSc, DDFS, and Subl3Rate, see Table 1*) are calibrated manually on catchment-averaged glacier mass balances derived from the IceSat dataset [67] for three upstream catchments: Hunza (Dainyor Bridge, UIB), Marshyangdi (Bimalnagar, UGB), and Sunkosh River (Wangdirapids, UBB). The IceSat dataset covers a 5-year period with an observation at the start of the period (i.e. October 2003) and an observation at the end of the period (i.e. September 2008). For the optimization of glacier and snow parameters, the model is run from October 2003 till September 2008, which is coinciding with the period that IceSat mass balances are available. The glacier mass balances resulting from the model runs are obtained by dividing the change in reported ice volumes over the entire run period 2003–2008 by the reported initial glacier area. Subsequently, a zonal average of the glacier mass balance is calculated for each upstream catchment, which is then used for calibration on the observed glacier mass balances. In addition to the calibration on glacier mass balances, simulated snow cover is compared with observed MODIS snow cover, which is derived from the MOD10CM dataset [65,66]. This comparison is performed on a monthly time step for the period March 2000 –December 2010, which is based on MODIS data availability. For each month, zonal averages are calculated of the MODIS snow cover imagery and the SPHY model snow cover output for each upstream catchment. Subsequently, the SPHY simulated snow cover is compared with the MODIS observed snow cover, and differences between the observed and simulated snow cover are minimized. The comparison between observed and simulated snow cover is needed for the optimization of the parameters related to snow accumulation and–melt. By obtaining the most optimal agreement between observed and simulated values for IceSat-derived glacier mass balances and MODIS-derived snow cover, snow parameters can be optimized.

After calibration of snow and glacier parameters, the parameters related to baseflow, lateral flow, and routing (*alphaGw, deltaGw, kx, GlacF, Rootdepth, and Subdepth, see Table 1*) are calibrated. Per basin, the simulated discharge is calibrated on time series of observed daily discharge from an up- and downstream gauging station. The following stations are used: Dainyor Bridge (Hunza, upstream UIB), Besham Qila (downstream UIB), Bimalnagar (Marshyangdi, upstream UGB), Devghat (downstream UGB), Wangdirapids (Sunkosh River, upstream UBB), and Sunkosh (downstream UBB) (see Fig 1). These gauging stations are selected based on data availability, and to have a representation of each of the upstream and downstream locations in each of the three river basins. The model is calibrated for the periods 2000–2005 (UIB and UGB), and 1998–2003 (UBB). The calibration and validation periods are selected based on the data availability in both the up- and downstream gauging stations. To optimize the performance of the model, the calibrated snow and glacier parameters are used to simulate snow storage over a 10-year period. The reported snow storage at the end of the 10-year period is subsequently used as initial snow storage in the calibration runs. In addition, a spin-up period of 3 years is used to initialize model states, such as soil moisture, snow storage, and groundwater. The model is calibrated using a random sampling technique with 50 different parameter combinations. From these combinations, the set is selected with the best performance, and corrected manually afterwards to optimize the model’s performance. After calibrating the model for the three upstream domains, the model is validated independently on different periods: 2008–2010 (UIB, Dainyor Bridge), 2006–2008 (UIB, Besham Qila), 2007–2009 (UGB), 2004–2008 (UBB).

### Future climate forcing

To account for the uncertainty in future climate change, an ensemble of downscaled General Circulation Model (GCM) runs is used to force the cryospheric-hydrological model. We select model runs from the medium stabilization scenario RCP4.5 and the very high baseline emission scenario RCP8.5 [70] to represent a wide range of possible futures. We did not include the mitigation scenario leading to a very low radiative forcing level (RCP2.6) as it is unlikely that this RCP can be met [71–73]. To meet RCP2.6 a drastic decline in carbon emissions is required, followed by ongoing carbon sequestration in the second half of the 21^st^ century [72]. It is however expected that the median of future cumulative carbon emissions will lie between RCP4.5 and RCP6.0 under current emission mitigation policies [73]. Moreover, future emissions from existing carbon-intensive industrial and energy capital are expected to remain large, limiting transformations to new capital that emits less carbon [71]. To aim for realistic projections, we therefore choose not to include RCP2.6 in the climate model ensemble. From the CMIP5 multi-model ensemble [74], we select four GCM runs for RCP4.5 and four GCM runs for RCP8.5 [20]. The GCM runs are selected in such way that they represent the full CMIP5 ensembles in terms of the projected ranges in means of future air temperature and precipitation, extremes of temperature and precipitation, and have sufficient skill over our region of interest [20].

The selected climate models are downscaled using the reference climate dataset, by applying the robust and well established Quantile Mapping methodology (e.g. [75,76]), which has been proven to perform well over mountainous regions [23,76]. We construct empirical cumulative density distributions (ecdfs) for each month of the year, at 5x5 km grid cells, from the daily values of the reference climate dataset and historical GCM runs for 1981–2010. These ecdfs are used to downscale and bias-correct future GCM runs spanning 2011–2100 at daily time step. We include frequency adaptation and the construction of new extremes. A detailed description of this approach has been published before [76]. In this way, transient hydrological model forcing series from 2011 until 2100 at 5x5 km spatial resolution and daily time step are constructed for each of the selected GCM runs.

### Analysis of climatic and hydrological extremes

We use the climate forcing and the outcomes of current and future model runs to analyze future changes in climatic and hydrological extremes. Changes in climatic extremes are evaluated for air temperature and precipitation by considering changes in several climatic indices. To characterize changes in air temperature (extremes) we analyze changes in the mean temperature and the warm spell days index (WSFI, hereafter HWFI) as defined by the European Climate Assessment Project (ECA)[77], which is the number of days in intervals of at least 6 days that the daily mean temperature is higher than the 90^th^ percentile of daily mean temperatures over a defined period. To characterize changes in precipitation (extremes) we analyze changes in the mean (annual) precipitation sum, the 95^th^ (P95) and 99^th^ (P99) percentiles of daily precipitation sums over a defined period, and the absolute maximum 5-day precipitation amount (RX5day) [77].

Changes in hydrological extremes are evaluated by focusing on high and low flow indices. For high flow, we analyze future changes in 99^th^ percentile of daily discharge levels, and the discharge levels of high flow events with a return period of 5, 25, and 50 years. The 5, 25, and 50-year return levels are calculated by determining the annual maximum flows, plotting the annual maximum flows by means of Gumbel plots [78], and calculating the discharge levels corresponding with events that occur once in 5, 25, and 50 years. In addition, we investigate future changes in annual maximum series (AMS) for the outlets of a rainfall-dominated basin (Brahmaputra) and a glacier/snow-melt dominated upstream basin (Hunza). Changes in the occurrence of low flows are analyzed by using flow duration curves (FDCs). To analyze these changes, we focus on the area between the 70 and 95 percentile exceedance discharge levels, which have a common use in the analysis of low flow frequencies [79,80].

## Results and discussion

### Calibration and validation

The calibration and validation of the SPHY model is performed by using a two-step systematic approach. The simulated mean snow cover and glacier mass balances resulting from the first calibration step are summarized in Table **2**. The simulated glacier mass balances match well with the observed glacier mass balances in all basins. Nevertheless, the mean snow cover is overestimated with 19–28%, where the largest overestimations occur in the Sunkosh basin. The overestimations in the snow cover may be attributed to the presence of cold biases in the temperature forcing, and to the fact that processes such as avalanching and snow re-distribution by wind were not considered in the model. This eventually may result in excessive accumulations of snow, and thus in the systematic overestimation of snow cover.

**Table 2:** Simulated and observed mean snow cover and glacier mass balance. Abbreviations: w.e. = water equivalent.

	Period	Hunza		Marshyangdi		Sunkosh	
		*Obs*	*Sim*	*Obs*	*Sim*	*Obs*	*Sim*
Mean Snow Cover [*%*]	2000–2010	56.6	81.0	26.7	46.1	19.2	47.6
Glacier Mass Balance [m w.e. y^-1^]	2003–2008	-0.08	-0.08	-0.21	-0.21	-0.23	-0.23

The best performing parameter sets resulting from the entire calibration approach are given in Table 3. Most calibrated snow and glacier parameters fall well within the range of those derived in other studies [9,14,81,82]. The calibrated snow parameter set in the UGB differs however from most calibrated values. For instance, the snow degree-day factor of 7.5 mm °C^-1^ day^-1^ is slightly higher than the range of 3–6 mm °C^-1^ day^-1^ that was found in other studies (e.g. [56]). Furthermore, the water storage capacity of the snow pack is lower than those that were found in previous work [9,14]. Only using these values, it was feasible to simulate a glacier mass balance similar to the IceSat observations. Lower snow degree-day factors would have resulted in positive glacier mass balances, which can likely be attributed to cold biases in the temperature forcing. Besides snow and glacier parameters, the calibrated rainfall-runoff parameters also fall in range of those reported in other studies [9,14,33].

**Table 3:** Calibrated model parameters and their values.

Parameters	Units	UIB	UGB	UBB
*Glacier*				
DDFG	mm °C day^-1^	6.3	8.5	5
DDFDG	mm °C day^-1^	3	6.5	3.5
*Snow*				
Tcrit	°C	0	0	0
SnowSc	mm mm^-1^	0.5	0.2	0.5
DDFS	mm °C day^-1^	6	7.5	4.1
Subl3Rate	mm day^-1^	1.5	3.2	1.25
*Rainfall—Runoff*				
alphaGW	-	0.005	0.062	0.005
deltaGW	day	1	10	1
Kx	-	0.955	0.93	0.96
GlacF	-	0.6	0.9	0.9
RootDepth	mm	800	653	600
SubDepth	mm	2766	2679	2705

The best performing calibration parameter datasets are used to simulate current and future discharge. Table 4 lists the results of the calibration and validation at six gauging stations. For the calibration period, the model shows a ‘satisfactory’ to ‘very good’ performance [83] with Nash-Sutcliffe Efficiency (NSE) values between 0.60 at Dainyor Bridge and 0.84 at Devghat. The biases between simulated and observed discharge are satisfactory at most gauging stations with biases up to 18% at Sunkosh station. At Dainyor Bridge the bias is large with a value of -31%. For the validation period, the model shows similar performances with NSE values between 0.62 at Besham Qila and 0.82 at Devghat. The biases are smaller in comparison with those reported for the calibration period with biases up to 16% at Dainyor Bridge. The Q90 biases, indicating the model’s performance under extreme flow conditions, show acceptable performances at the downstream located gauging stations with biases up to 12%, whereas at the upstream located gauging stations the biases are larger with values between -24% and -30%. The underestimation in the extreme flows and the flow at Dainyor Bridge (i.e. during the calibration period) as well are likely a consequence of the underestimate in high-mountain precipitation, and overestimated snow cover, which eventually may lead to an underestimation of the discharge due to lower fractions of direct runoff. Moreover, the spatial resolution of the model (i.e. 5 x 5 km) might be a reason for the underestimations in the peak flows. Higher spatial resolutions would be more favorable for routing and could lead to better results, but is a large computational expense.

**Table 4:** Model performance ratings in terms of NSE and PBIAS that were calculated on daily basis for the calibration and validation periods, separately. **PBIAS Q90 represents the percent bias for the 90-percentile discharge level for the calibration and validation periods.** Abbreviations: C = Calibration, V = Validation.

	NSE C	NSE V	PBIAS C	PBIAS V	PBIAS Q90 C & V
Dainyor Bridge	0.60	0.74	-30.9	-16.0	-23.7^1^
Besham Qila	0.69	0.62	-9.2	2.6	8.8^2^
Bimalnagar	0.78	0.75	-10.0	-12.9	-29.7^3^
Devghat	0.84	0.82	1.8	3.1	-12.2^3^
Wangdirapids	0.76	0.75	-4.0	-8.7	-24.0^4^
Sunkosh	0.68	0.74	17.5	8.2	-0.6^4^

^1^ 2000–2010

^2^ 2000–2008

^3^ 2000–2009

^4^ 1998–2008

**Fig 3:**
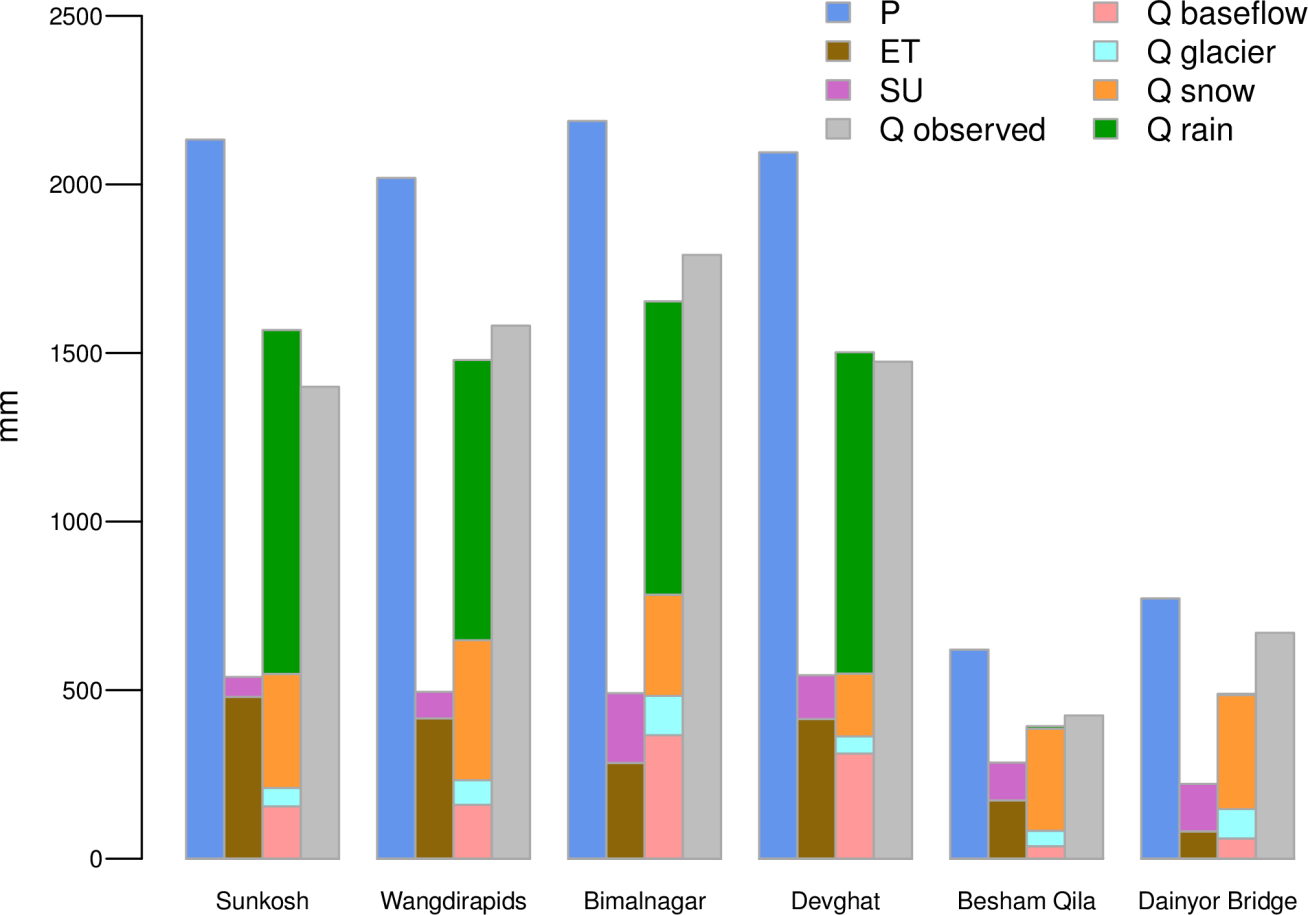
Annual catchment-averages of most important water balance components and observed discharge at 6 gauging stations used for calibration and validation. Abbreviations: P = precipitation, ET = actual evapotranspiration, SU = actual sublimation, Q observed = observed discharge, Q baseflow = baseflow, Q glacier = glacier melt, Q snow = snow melt, and Q rain = rainfall runoff.

The annual water balances for 1998–2010 (i.e. coinciding the period between the first calibration year and the last validation year) show negligible gaps ranging 1–4 mm yr^-1^ (see Fig 3). The gaps can be attributed to changes in the storages of snow, soil, and groundwater reservoirs. Given the uncertainties in the meteorological forcing over high mountain terrain and the limitation in feasible model resolutions for simulating such large areas, we conclude that the model’s performance is sufficient for the analysis of hydrological extremes.

### Future climate change

Towards the end of the 21^st^ century both selected RCP4.5 and RCP8.5 model runs indicate that both precipitation and temperature will increase in magnitude. Table 5 lists the basin-averaged values for several climate variables under reference climate conditions and their projected relative (precipitation) and absolute (temperature) changes under far future climate conditions (2071–2100). Under present climate conditions, both, the annual precipitation sums and the precipitation extremes are generally highest in the UGB. Only the present maximum 5-day precipitation amounts are highest in the UIB. All climate models indicate, in general, that future precipitation amounts and extremes will increase in all domains, where the relative changes in precipitation extremes are projected to be higher than the relative changes in annual precipitation sums. Climate models representing wet climate conditions project in general the largest relative increases in future annual precipitation sums with increases up to 18% and 56% in the UIB and UBB, respectively, under RCP8.5. In the UGB, the largest increases are projected by cold/dry models under RCP8.5 (i.e. inmcm4) with a relative increase of 41%. In terms of precipitation extremes, a consistent pattern can be observed with cold/wet climate models (i.e. bcc-csm) and warm/wet climate models (i.e. CanESM2) projecting the largest relative changes in the UIB and UBB, respectively, under RCP8.5. The same pattern can also be recognized for the projected relative changes in annual precipitation sums. In the UGB, the pattern is less consistent. Two precipitation extreme indices (P90 and RX5day) are projected to change most under warm/wet (RCP 4.5) climate conditions. P95 and P99 are projected to change most under cold/dry (RCP8.5) and warm/wet (RCP8.5) climate conditions, respectively. In addition to the projected increases in precipitation amounts and extremes, the mean air temperature is also projected to increase towards future, where the largest increases are projected under warm (RCP8.5) climate conditions with temperature increases in the range 4.8–5.6°C among the different basins. Significant increases in the HWFI are projected by models characterizing warm climate conditions, where the largest increases are projected by the CMCC-CMS model under RCP4.5. Smaller increases are projected by models characterizing cold climate conditions, where the most limited change is predicted by the BNU-ESM model.

**Table 5:** Basin-averaged values for a range of climate variables for the reference period and the relative (precipitation) and absolute (temperature) changes at the end of the 21^st^ century as projected by each of the downscaled GCM runs used in this study. Abbreviations: P = mean annual precipitation sum, P90, P95, and P99: 90^th^, 95^th^, 99^th^ percentiles of daily precipitation sums, RX5 = maximum 5-day precipitation amount, T = annual mean temperature, and HWFI = warm spell days index.

		1981–2010	2071–2100							
			RCP4.5				RCP8.5			
Climate variable	Basin	Reference	BNU-ESM r1i1p1	inmcm4 r1i1p1	CMCC-CMS r1i1p1	CSIRO-Mk3-6-0 r4i1p1	inmcm4 r1i1p1	CMCC-CMS r1i1p1	bcc-csm1-1 r1i1p1	CanESM2 r3i1p1
P (mm yr^-1^)	UIB	1013	+21%	-1%	+4%	+18%	+17%	+3%	+18%	+14%
	UGB	1811	+21%	+2%	+1%	+20%	+41%	-5%	+28%	+34%
	UBB	1483	+15%	-2%	+6%	+7%	+29%	+12%	+23%	+56%
P_90_ (mm d^-1^)	UIB	15.1	+31%	+21%	+56%	+53%	+42%	+55%	+87%	+58%
	UGB	15.8	+17%	+10%	+30%	+68%	+66%	+23%	+62%	+54%
	UBB	12.8	+12%	-1%	+11%	+15%	+28%	+18%	+20%	+52%
P_95_ (mm d^-1^)	UIB	18.1	+25%	+14%	+39%	+40%	+29%	+40%	+66%	+43%
	UGB	23.2	+20%	+3%	+12%	+38%	+52%	+4%	+33%	+41%
	UBB	18.4	+16%	+1%	+9%	+12%	+30%	+18%	+29%	+61%
P_99_ (mm d^-1^)	UIB	36.8	+26%	+5%	+23%	+33%	+30%	+27%	+40%	+19%
	UGB	46.5	+34%	+10%	+11%	+43%	+62%	+11%	+47%	+68%
	UBB	35.2	+24%	+3%	+23%	+15%	+30%	+50%	+43%	+104%
RX5day (mm)	UIB	352	+55%	+26%	+52%	+72%	+51%	+59%	+108%	+39%
	UGB	325	+58%	+113%	+76%	+170%	+131%	+58%	+122%	+123%
	UBB	245	+61%	+40%	+71%	+32%	+107%	+87%	+70%	+115%
Mean air T (°C)	UIB	-1.9	+1,7	+0,6	+2,7	+3,1	+2,6	+5,3	+3,6	+5,6
	UGB	3.2	+1,5	+0,7	+2,5	+2,8	+2,5	+4,8	+3,0	+4,1
	UBB	-0.8	+1,5	+0,7	+2,7	+3,0	+2,6	+5,0	+3,3	+4,5
HWFI (d yr^-1^)	UIB	9	0	+104	+185	+28	+24	+103	+58	+107
	UGB	11	+2	+104	+182	+38	+69	+146	+83	+108
	UBB	12	0	+105	+178	+35	+65	+132	+70	+113

**Fig 4:**
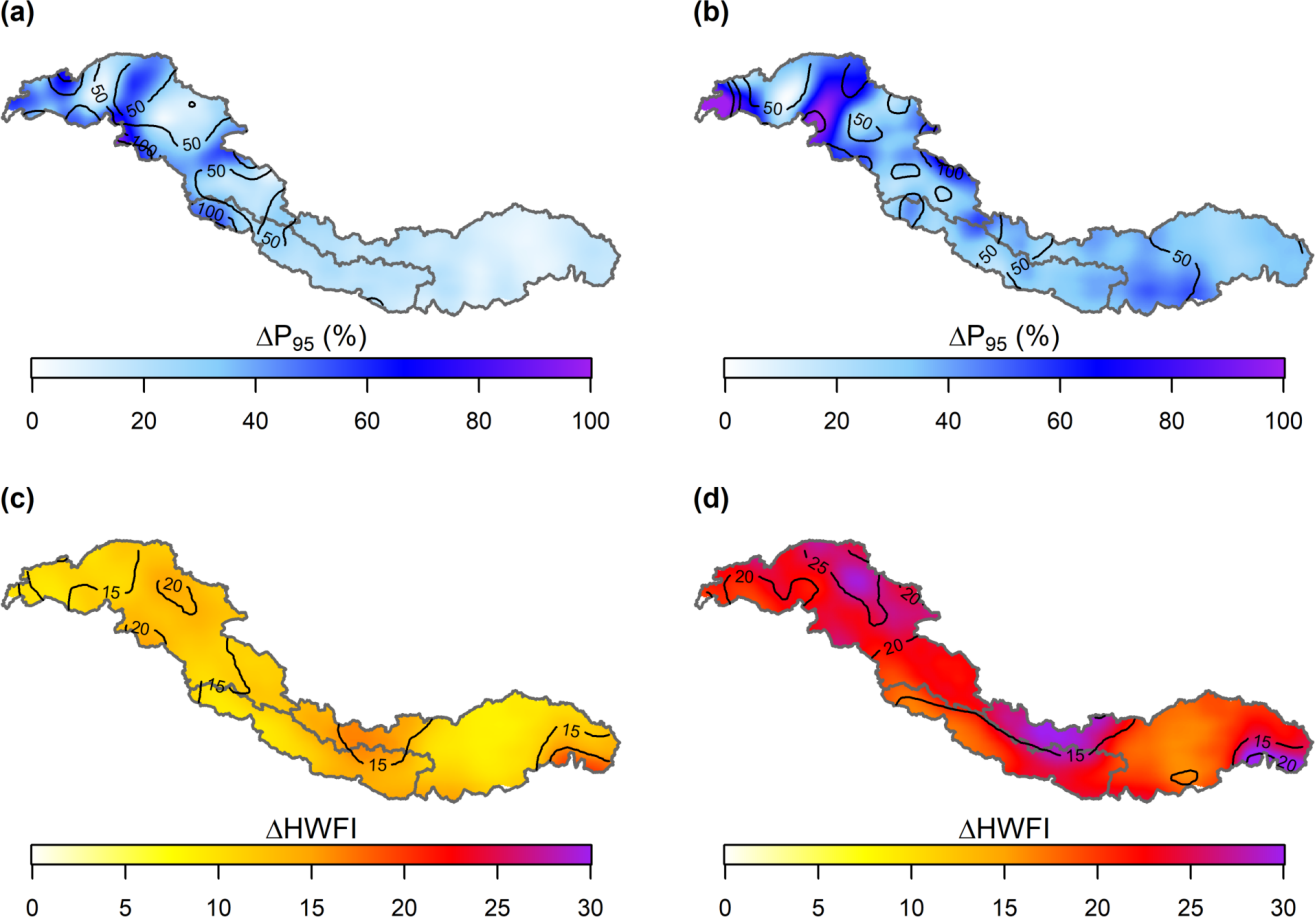
Changes in P95 and HWFI. Maps showing the changes in P95 and the HWFI index for RCP4.5 and RCP8.5. Contour lines denote the ensemble range of projections.

Fig 4 shows the mean magnitude of change in precipitation (i.e. P95) and temperature (i.e. HWFI) extremes that is expected to occur under RCP4.5 and RCP8.5. Relative changes in P95 are generally greatest in the UIB with relative increases of more than 100% at the southern margins of the UIB under RCP4.5 and relative increases up to about 130% at the westernmost border of the UIB under RCP8.5. These relative increases can mainly be attributed to increases in P95 that are projected by all models characterizing warm and wet climate conditions. A pattern can be recognized with the largest increases in the western part of the IGB and smaller increases when moving eastward. This pattern can mainly be attributed to the projected changes that result from warm and wet climate models. The HWFI is projected to increase with a factor up to about 20 in parts of the UIB, the northern part of the UBB, and the southernmost margins of the UBB under RCP4.5, and with a factor of up to 40 at the southernmost margins of the UBB under RCP.8.5. These increases are mainly deriving from model runs characterizing warm climate conditions. Both, the relative changes in P95 and HWFI, are accompanied with large spreads in those regions where the greatest changes are projected, which indicates that these changes have a large uncertainty.

### Future changes in hydrological extremes

#### High flows

**Fig 5:**
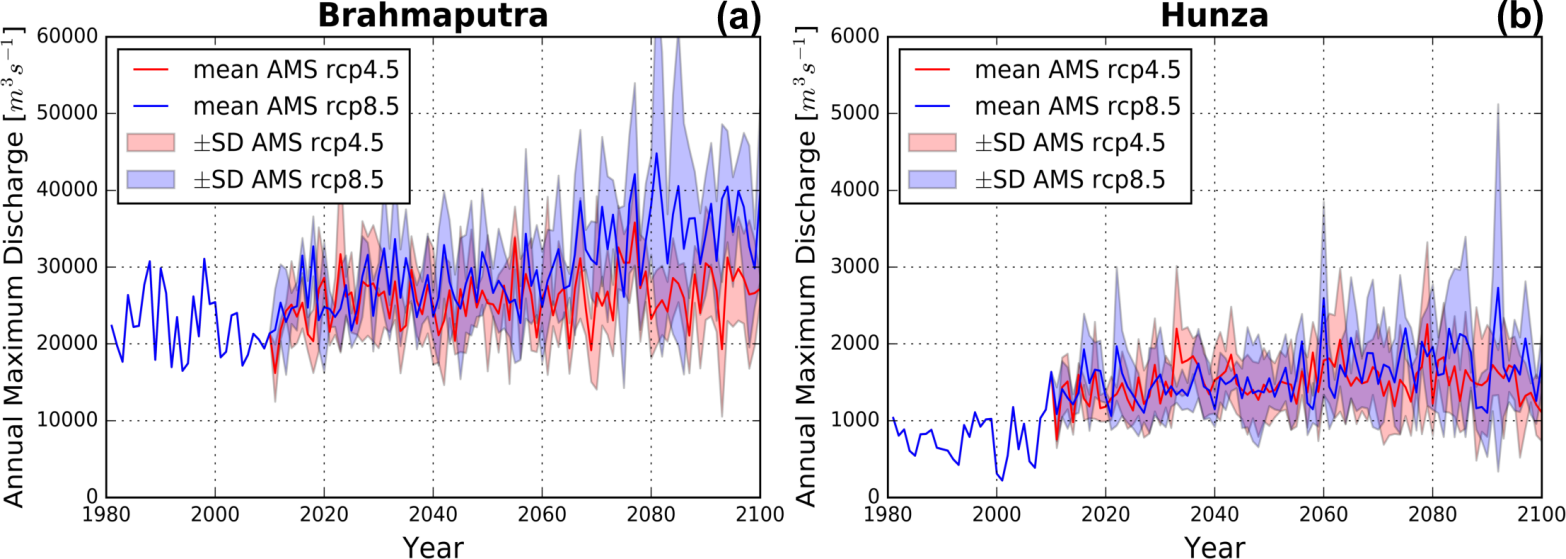
Present and future annual maximum series (AMS). The mean AMS for the period 1981–2100 under RCP4.5 (red) and RCP8.5 (blue). The AMS are given for the Upper Brahmaputra and Hunza basins. The colored band represents the standard deviation resulting from forcing the hydrological model with the different climate models.

Based on the projected trends in precipitation amounts and extremes it can be expected that high flow conditions will occur more frequently in the IGB towards future. Fig 5A and 5B show the future projections in annual maximum series (AMS) for the outlets of the rainfall-dominated UBB and the glacier/snow-melt dominated Hunza basin (UIB) under RCP4.5 and RCP8.5 scenarios. Relative to the reference period (1981–2010) there is a significant increase in AMS in both basins and under both scenarios. In the Hunza basin the differences between RCP4.5 projections and RCP8.5 projections are relatively small, whereas in the UBB these differences are larger with a more significant increase in AMS under RCP8.5. The AMS increases in the Hunza basin can mainly be attributed to increases in snowmelt under RCP4.5 and a combination of increases in snowmelt and rainfall under RCP8.5. Since the increases in the HWFI and P95 are considerable in the UIB, especially under RCP8.5 (see Fig 4), it is likely that increasing temperature and precipitation extremes under RCP4.5 and RCP8.5 may contribute to increasing AMS. In the UBB increasing precipitation extremes under RCP4.5 and RCP8.5 may contribute to increasing AMS. The P95 increases are not as large as in the UIB, and the HWFI increases are considerable (see Fig 4), especially at the southernmost margins of the UBB where the Himalayas merge into the Indo-Gangetic plains. Nevertheless, HWFI increases are largest in those regions that are not or limited covered by snow and ice, which means precipitation extremes might be interpreted as the main responsible factor in AMS increases. Although the AMS increases are larger in the UBB, it can also be observed that the standard deviation becomes larger towards the end of the 21^st^ century. A similar trend can be observed in the Hunza basin, which indicates a larger uncertainty in future AMS trends.

Increasing high flow trends have also been projected in other basins. In Table 6 the mean discharge, the 99^th^ percentile of daily discharge values (Q1), and 5, 25, and 50-year return levels under present, near future (2035–2064), and far future (2071–2100) RCP4.5 and RCP8.5 climate conditions are listed for the outlets of the UIB, UGB, UBB, Hunza, Marshyangdi, and Sankosh basins. The highest mean discharge values are simulated at the outlet of the UBB with a mean rate of 6120 m^3^s^-1^ and is projected to change with relative increases up to 49% at the end of the 21^st^ century. The lowest mean discharge values are simulated at the outlet of the Hunza basin with a mean rate of 160 m^3^s^-1^ and is projected to change with relative increases up to 119% under RCP8.5, which will end up in a higher projected discharge rate than in another upstream catchment, the Marshyangdi basin (i.e. 350 m^3^s^-1^ in the Hunza basin vs. 283 m^3^s^-1^ in the Marshyangdi basin). The higher projected discharge rates can be explained by the increased snowmelt in the Hunza basin, whereas in the Marshyangdi basin snowmelt is projected to increase initially followed by a decline after 2040–2050. In addition to the mean discharge rates, flow extremes are also expected to increase in magnitude towards future. The largest increases are projected in the Sunkosh basin, where the 50-year return level is expected to increase with 147% after 2071 under RCP8.5. In the UIB basin, the smallest increases are projected. In this basin, the 50-year return level is projected to increase with 51% under RCP8.5. Accompanied with higher discharge levels, standard deviations and thus uncertainties also increase.

**Table 6:** Changes in the mean discharge, 99^th^ percentile of daily discharge values (Q99), and the discharge levels of events with return periods of 5, 25 and 50 years at the outlets of the UIB, UGB, UBB, and the upstream catchments (Hunza, Marshyangdi, and Sankosh) under present (1981–2010), near future (2035–2064), and far future (2071–2100) RCP4.5 and RCP8.5 climate conditions. The values between the parentheses represent the standard deviation.

Basin	Period	Units	$\bar{Q}$	Q99	5-yr Q	25-yr Q	50-yr Q
Hunza	1981–2010	m^3^s^-1^	160	972	1045	1500	1689
	2035–2064 RCP4.5	%	+102 (39)	+93 (33)	+87 (34)	+76 (35)	+73 (35)
	2071–2100 RCP4.5	%	+88 (32)	+96 (51)	+89 (41)	+86 (44)	+85 (44)
	2035–2064 RCP8.5	%	+101 (30)	+86 (26)	+80 (34)	+70 (36)	+67 (37)
	2071–2100 RCP8.5	%	+119 (34)	+121 (27)	+118 (30)	+116 (31)	+116 (32)
Marshyangdi	1981–2010	m^3^s^-1^	199	884	1018	1275	1381
	2035–2064 RCP4.5	%	+16 (10)	+35(15)	+40 (17)	+53 (26)	+56 (29)
	2071–2100 RCP4.5	%	+28 (16)	+62 (23)	+63 (26)	+77 (30)	+81 (31)
	2035–2064 RCP8.5	%	+21 (11)	+54 (19)	+56 (15)	+74 (14)	+79 (14)
	2071–2100 RCP8.5	%	+42 (29)	+93 (53)	+96 (51)	+114 (59)	+120 (61)
Wangdirapids	1981–2010	m^3^s^-1^	288	1167	1176	1511	1650
	2035–2064 RCP4.5	%	+18 (14)	+28 (17)	+31 (18)	+36 (20)	+37 (21)
	2071–2100 RCP4.5	%	+29 (12)	+45 (17)	+46 (21)	+52 (22)	+54 (22)
	2035–2064 RCP8.5	%	+28 (15)	+40 (16)	+41 (20)	+46 (21)	+47 (22)
	2071–2100 RCP8.5	%	+66 (40)	+107 (73)	+121 (76)	+141 (92)	+147 (97)
Upper Indus	1981–2010	m^3^s^-1^	2177	13063	15281	21659	24301
	2035–2064 RCP4.5	%	+59 (25)	+54 (28)	+42 (18)	+39 (16)	+39 (16)
	2071–2100 RCP4.5	%	+49 (17)	+55 (32)	+48 (29)	+51 (32)	+52 (33)
	2035–2064 RCP8.5	%	+50 (11)	+46 (24)	+28 (28)	+24 (28)	+23 (28)
	2071–2100 RCP8.5	%	+51 (11)	+59 (29)	+47 (19)	+50 (21)	+51 (21)
Upper Ganges	1981–2010	m^3^s^-1^	1536	6639	7373	9015	9695
	2035–2064 RCP4.5	%	+16 (11)	+36 (15)	+38 (16)	+53 (24)	+57 (27)
	2071–2100 RCP4.5	%	+29 (17)	+60 (22)	+60 (29)	+75 (34)	+80 (35)
	2035–2064 RCP8.5	%	+20 (11)	+52 (18)	+49 (14)	+68 (14)	+74 (14)
	2071–2100 RCP8.5	%	+41 (31)	+83 (47)	+84 (53)	+102 (64)	+108 (68)
Upper Brahmaputra	1981–2010	m^3^s^-1^	6120	25495	25949	32242	34847
	2035–2064 RCP4.5	%	+16 (16)	+15 (10)	+21 (10)	+26 (8)	+28 (7)
	2071–2100 RCP4.5	%	+24 (11)	+21 (5)	+32 (6)	+39 (8)	+41 (10)
	2035–2064 RCP8.5	%	+24 (14)	+26 (17)	+29 (18)	+33 (20)	+34 (20)
	2071–2100 RCP8.5	%	+49 (33)	+57 (35)	+68 (36)	+77 (39)	+80 (39)

**Fig 6:**
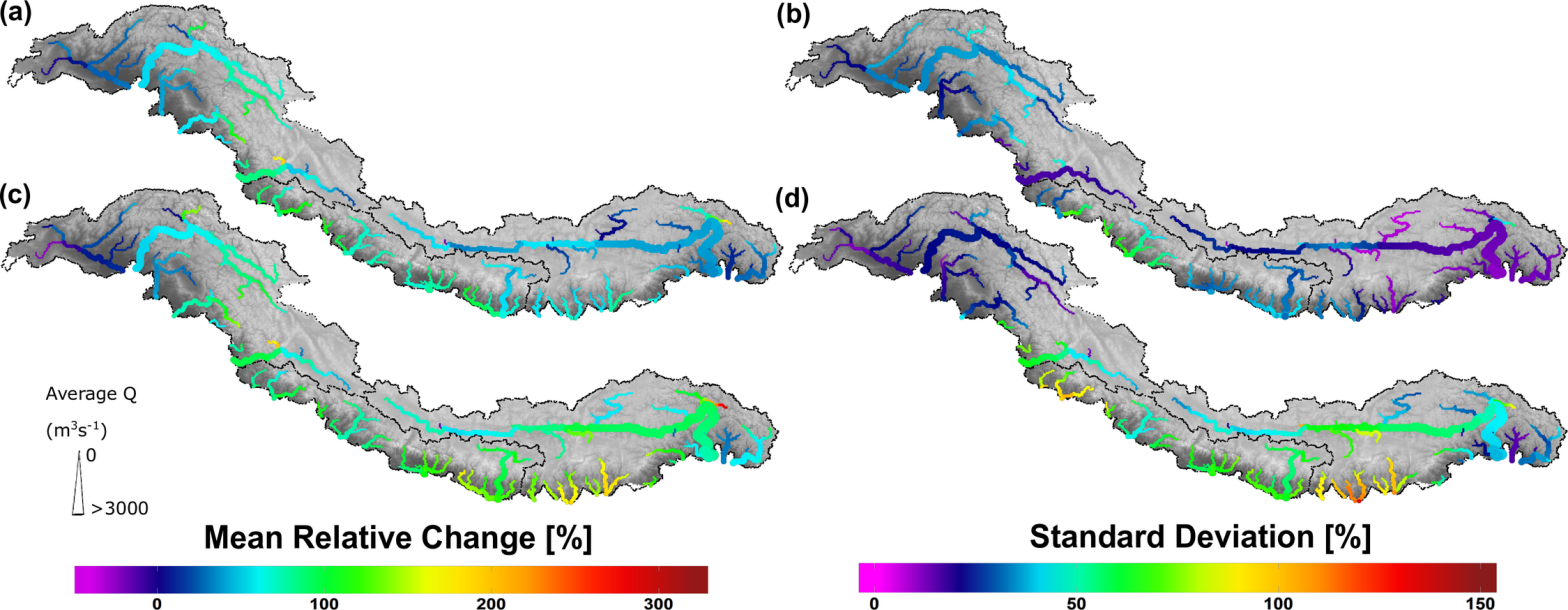
Relative changes in 50-year return period discharge level. Maps showing the mean relative changes (left) in 50-year return period discharge levels and their standard deviations (right) at the end of 21^st^ century (2071–2100) under RCP4.5 (upper row) and RCP8.5 (lower row) scenarios.

Fig 6 shows the mean and the standard deviation of the relative change in the 50-year return period over the river network under RCP4.5 and RCP8.5 scenarios. In the most parts of the IGB the 50-year return level is projected to increase with relative increases up to about 305% under RCP8.5 climate conditions. The largest increases are projected for the easternmost upstream headwaters of the Brahmaputra. These increases can mainly be attributed to increases in rainfall resulting from increases in precipitation, and partly also to increases in ice melt. Besides increases, there are also river branches where the 50-year return level is projected to decrease. These decreases (i.e. up to about 25%) mainly occur in the westernmost part of the UIB (i.e. Kabul basin) and can mainly be attributed to decreases in ice and snowmelt, and precipitation as well. Similar trends were also projected by a previous study [14] in the UIB. The standard deviation of 50-year return levels is generally larger under RCP8.5 (see Fig 6D) with the largest deviations at the southern margins of the UBB. In this region, also relatively large changes are projected with relative increases up to about 200%.

The projected changes in high flow characteristics are in line with the reported trends in other studies that were conducted in the Indus, Ganges and/or Brahmaputra basins [13,14,37,41]. Although the trends are similar, it is difficult to compare the magnitude of absolute and relative changes in discharge levels with those projected in other studies. The underlying reason is that other studies have used different climate forcing and approaches to investigate impacts of climate change on hydrological extremes. Furthermore, different locations hamper comparisons between our study and other studies. For instance, most studies have focussed on the entire Indus, Ganges or Brahmaputra basins, whereas in our study the focus is on the upstream mountainous domains. Finally, different time periods may hamper the comparisons. Although there is a high degree of similarity between the periods used for comparing far future changes (e.g. [13]), the periods used for near future changes can differ. For example, one of the referred studies [14] defined 2021–2050 as a near future period, whereas we defined 2035–2064 as a near future period.

#### Low Flows

**Fig 7:**
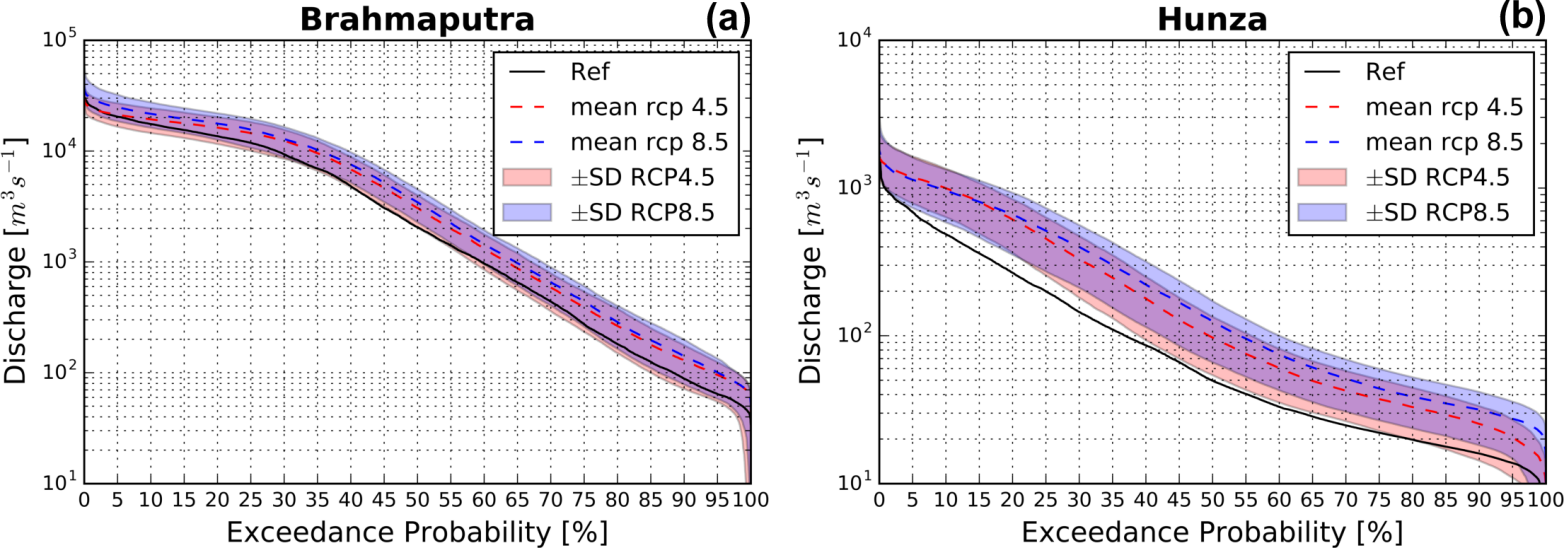
Reference and future flow duration curves (FDCs). The FDCs of the reference (black lines) and far future period (dashed lines) at the outlets of the Upper Brahmaputra and Hunza basins. The FDCs represent mean flow conditions under RCP4.5 (red) and RCP8.5 (blue). The colored band represents the standard deviation resulting from forcing the hydrological model with the different climate models.

Low flows are in general projected to occur less frequently. In Fig 7 the flow duration curves (FDCs) are given for the outlets of the Hunza basin and the UBB under reference climate conditions, and RCP4.5 and RCP8.5 scenarios. Focusing on the area between the 70 and 95 percentile exceedance discharge levels, it can be observed that low flow conditions are in general projected to occur less frequently in the UBB under both RCP4.5 and RCP8.5. Previous work [38,41] projected similar trends for the Brahmaputra. In the Hunza Basin, a similar trend can be observed, although the uncertainty is quite large. These uncertainties can likely be attributed to the large spread among the different climate models. Both in the UBB and the Hunza basin there are a few climate models (i.e. mainly cold/dry climate models (inmcm4)) that project a slightly higher frequency in low flow conditions, especially for RCP4.5 in the Hunza basin. This may explain why the increase in the far future mean discharge in the Hunza basin is projected to be lower than the increase in the near future mean discharge for RCP4.5 (see Table 6), and can be attributed to a decline in glacier melt after 2050.

### Uncertainties and limitations

The projections of future hydrological extremes are subject to several uncertainties and limitations that are briefly discussed below. Although significant trends for the future have been identified, the outcomes of this study should be treated with care. The uncertainties and limitations are mainly related to the input data, the climate projections, the representation of physical processes in the hydrological model, and the parameterization of the model or are emerging from the natural variability of climate variables.

In this study, input data, such as meteorological observation data, was used to force the hydrological model or to conduct bias-corrections on the reference climate dataset. As mentioned before, there were 40 meteorological data records available that are unequally distributed over the study area, and are valley-oriented. This means in many regions, and especially in the high-altitude areas, there is a lack of measurement data. Consequently, uncertainties are introduced when conducting station-based bias-corrections on temperature fields. The uncertainties in the reference climate dataset are subsequently introduced in the future climate forcing since the reference climate dataset was used to downscale different GCM runs.

The future climate forcing does consist of 8 climate models, each representing different climate conditions, under the RCP4.5 and RCP8.5 emission scenarios. The models that were used for this study were selected according to an advanced envelope-based selection procedure [20], comprising three steps focusing on changes in climatic means and extremes, and the skill to simulate present and/or historical climate. The selection approach is decisive for which models are selected. Another selection approach may result in the selection of different climate models and thus in different projections of future hydrological extremes. The climate models selected in this study were evaluated for their projected changes in climatic extremes in an earlier published study [20]. In general, there is a consensus between the selected models on the projected trends in temperature and precipitation extremes. Nevertheless, the spread among the different models can be large. For instance, in the UBB the projected increases in P99 under RCP8.5 can vary from +30% under cold/dry climate conditions (i.e. inmcm4, see Table 5) to +104% under warm/wet climate conditions (i.e. CanESM2). In addition, the spatial spread differences can be large. For example, the spread in relative P95 changes in the UIB (see Fig 4) can exceed 100%, whereas in the UBB the spread can be smaller than 50%, meaning that there is less consensus about the projected changes in the UIB than in the UBB. Furthermore, it may happen that different climate models project the largest changes in different precipitation indices among the different basins. In the UIB and UBB, the largest changes are projected by cold/wet and warm/wet climate (models, respectively (see Table 5), whereas in the UGB the largest changes are projected by both warm/wet and cold/dry models. The missing consensus among the different climate models indicate that further improvement is needed in the representation of climate extremes in GCMs.

The SPHY model was used for the simulation of present and future daily discharge. Within the SPHY model the representation of physical processes, such as the simulation of snow processes, introduces additional uncertainties. The SPHY model can simulate snow melt, accumulation, refreezing, and sublimation processes, but does not take processes such as gravitational snow transport or snow re-distribution by wind into account. This may eventually result in an overestimation of snow storage and cover. To overcome these uncertainties, snow transport models, such as SnowSlide [84], can be integrated at higher spatial resolutions of modelling to simulate e.g. gravitational snow transport by avalanching. In addition, uncertainties can be introduced by simplified model assumptions. For instance, snow sublimation is modelled by a simple elevation-dependent potential sublimation function [14], thereby assuming that sublimation is constant over time and that most sublimation occurs at higher altitudes where the highest wind speeds prevail and air is driest. Nevertheless, sublimation varies in time, due to its dependency on wind speed and humidity, amongst others. To account for this temporal variability, it may be considered to use more sophisticated approaches including energy balance components. However, these approaches require more data, which is often limited available or even absent in the remote areas of the IGB. Furthermore, future glacier change and melt projections are associated with limitations in the improved glacier module. The module is less accurate for very large model resolutions. At large resolutions, the small glaciers fall within one grid cell, which disables the possibility for re-distributing ice over these glaciers. Another disadvantage is that glaciers cannot increase in area, which disables the possibility to simulate glacier surges. Another uncertainty emerges from the differentiation of debris-free and debris-covered glaciers. The differentiation is based on thresholds of elevation and slope without consideration of local geology and geomorphology. This may affect the differentiation of glaciers and subsequently the amount of ice melt that can be produced from the glaciers. The problem, however, is that the knowledge about local geology and geomorphology is limited in the HKH region, and no glacier inventory, making a distinction in debris-covered and debris-free glaciers for the entire HKH, is available.

The calibration of the SPHY model resulted in a uniform parameter set for each river basin specifically. Uncertainties are introduced since the values of most parameters vary in space and time. To reduce the uncertainties related to the spatial variability in parameter values one may consider subdividing each domain in smaller sub-catchments and to use regionalisation approaches. One of the regionalisation approaches includes a similarity approach in which parameter sets that are calibrated for gauged catchments are transposed to ungauged catchments with similar climatic and physiographic characteristics (e.g. topography, land use, soils, geology, stream network, etc.). A study to different regionalisation approaches in the Austrian Alps showed that similarity approaches and kriging approaches belong to the best performing regionalisation methods [85]. Regionalisation approaches have recently also been applied in the HKH region. In a recent study a similarity approach was conducted in two glacierized subcatchments in the Koshi catchment, Nepal (i.e. located in the eastern part of the IGB) [86]. The outcomes of the cited study indicated that the transfer of calibrated parameters from a gauged catchment to a neighbouring ungauged catchment is viable and that the use of regionalisation approaches has potential in other ungauged catchments in the Himalayan region. It is however difficult to implement regionalisation approaches in the entire HKH region, since a lot of detailed information (e.g. on geology) required for the implementation of regionalisation approaches is lacking. Our approach in assigning parameter sets can be considered as a regionalisation because we transfer parameter sets from gauged catchments within a river basin to ungauged parts of the basin, for the UIB, UGB, and UBB separately. Other parameter uncertainties might emerge from the over-parameterization of parameters or the appearance of inter-correlation between parameters [87]. Since the calibrated snow degree-day factor in the Marshyangdi basin is higher than the range of 3–6 mm °C^-1^ day^-1^ found in other studies (see Table 3) it might be concluded that this parameter is over-parameterized.

This study focussed on the propagation of uncertainties in future climate (i.e. the spread in climate projections) and future hydrological projections and did not focus on uncertainties, such as parameter uncertainties in detail, since this focus is beyond the scope of this work. To have a full impression of the uncertainties a full uncertainty analysis is recommended for future work.

## Conclusions

The aim of this study is to investigate the impacts of climate change on hydrological extremes in the upstream domains of the Indus, Ganges, and Brahmaputra. To this end, we use the fully distributed cryospheric-hydrological SPHY model to simulate current and future daily discharge. The model is forced by bias-corrected and downscaled GCM runs that represent different future climate conditions under RCP4.5 and RCP8.5. The climate forcing and the outcomes of the models are used to analyse climatic and hydrological extremes (i.e. high and low flow extremes).

Climatic extremes are projected to increase in magnitude towards the end of the 21^st^ century. Thereby, the increases in climatic extremes are projected to be stronger than the increases in climatic means. The magnitude of the absolute and relative changes in temperature and precipitation extremes and the regions where these changes occur depend highly on which climate conditions will prevail. In general, it can be concluded that precipitation extremes (i.e. P95) will increase mostly in the Upper Indus Basin with relative increases up to 130%. Temperature extremes are expected to appear more frequently in the future, where the HWFI is projected to increase with a factor up to 40 at the southern margins of the Upper Brahmaputra Basin.

The outcomes indicate further that mean discharge and high flow conditions will increase towards future. In rainfall-dominated basins as the UBB increases in precipitation extremes may contribute in discharge extremes. To which extent precipitation and temperature extremes might contribute to increases in discharge extremes in glacier/snowmelt-dominated basins depend on the magnitude of changes in extremes. In case of the Hunza basin, both temperature and precipitation extremes might contribute to increasing discharge extremes due to increasing temperature (i.e. HWFI) and precipitation (P95) indices. In general, an increase in mean discharge, the 99^th^ percentile, and the 5, 25, and 50-year return levels is expected in all basins. The 50-year return level is expected to increase up to 305% relative to the current level with the largest increases in the upstream headwaters of the Upper Brahmaputra basin. In the westernmost part of the Upper Indus basin, the 50-year return level is expected to decrease up to 25%. These changes can be attributed to changing contributions of rainfall, ice and snowmelt. In the upstream headwaters of the UBB rainfall increases are mainly responsible for the changes in the 50-year return level, which is mainly a consequence of increasing precipitation. In addition, increases in ice melt also contribute to these changes. In the westernmost part of the UIB precipitation, ice and snowmelt decreases are mainly responsible for changes in the 50-year return level. Low flows are in general projected to occur less frequently in the Upper Brahmaputra and Hunza basins. Nevertheless, the uncertainty of low flow projections in the Hunza basin is high.

The outcomes of this study aim to contribute to a better understanding on the impacts of climate change on hydrological extremes in the HKH region. The outcomes may contribute to the development of adaptation strategies to reduce the adverse impacts of changes in climatic and hydrological extremes. The outcomes are sufficiently reliable to extract main trends, but are also subject to many uncertainties, which means the outcomes should be treated with care and improvements are needed in future research on hydrological extremes.
